# Effects of different pedicle screw insertion depths on sagittal balance of lumbar degenerative spondylolisthesis, a retrospective comparative study

**DOI:** 10.1186/s12891-021-04736-1

**Published:** 2021-10-05

**Authors:** Quan Zhou, Jun-xin Zhang, Yi-fei Zheng, Yun Teng, Hui-lin Yang, Hao Liu, Tao Liu

**Affiliations:** 1 Department of Orthopaedics, The First Affiliated Hospital of Soochow University, 899 Pinghai Road, Suzhou, 215006 China; 2 The Affiliated Suzhou Science & Technology Town Hospital of Nanjing Medical University, No. 1, Lijiang Road, Suzhou, 215006 Jiangsu China

**Keywords:** Sagittal balance, Spino-pelvic parameters, Lumbar spondylolisthesis, Degenerative disease of the spine, Posterior lumbar interbody and fusion, Insertion depth

## Abstract

**Background:**

Few reports to date have evaluated the effects of different pedicle screw insertion depths on sagittal balance and prognosis after posterior lumbar interbody and fusion (PLIF) in patients with lumbar degenerative spondylolisthesis (LDS).

**Methods:**

A total of 88 patients with single-level PLIF for LDS from January 2018 to December 2019 were enrolled. Long screw group (Group L): 52 patients underwent long pedicle screw fixation (the leading edge of the screw exceeded 80% of the anteroposterior diameter of vertebral body). Short screw group (Group S): 36 patients underwent short pedicle screw fixation (the leading edge of the screw was less than 60% of the anteroposterior diameter of vertebral body). Local deformity parameters of spondylolisthesis including slip degree (SD) and segment lordosis (SL), spino-pelvic sagittal plane parameters including pelvic incidence (PI), pelvic tilt (PT), sacral slope (SS) and lumbar lordosis (LL), Oswestry Disability Index (ODI), and Visual Analog Scale (VAS) for back pain of both groups were compared. Postoperative complications, including vertebral fusion rate and screw loosening rate, were recorded.

**Results:**

Except that PI in Group S at the final follow-up was not statistically different from the preoperative value (*P* > 0.05), other parameters were significantly improved compared with preoperative values one month after surgery and at the final follow-up (*P <* 0.05). There was no significant difference in parameters between Group L and Group S before and one month after surgery (*P* > 0.05). At the final follow-up, SD, SL, LL, PT and PI-LL differed significantly between the two groups (*P <* 0.05). Compared with the preoperative results, ODI and VAS in both groups decreased significantly one month after surgery and at the final follow-up (*P <* 0.05). Significant differences of ODI and VAS were found between the two groups at the final follow-up (*P <* 0.05). Postoperative complications were not statistically significant between the two groups (*P >* 0.05).

**Conclusions:**

PLIF can significantly improve the prognosis of patients with LDS. In terms of outcomes with an average follow-up time of 2 years, the deeper the screw depth is within the safe range, the better the spino-pelvic sagittal balance may be restored and the better the quality of life may be.

**Supplementary Information:**

The online version contains supplementary material available at 10.1186/s12891-021-04736-1.

## Background

Degenerative diseases of the spine are caused by the gradual degeneration of the discs with increasing age, most often involving the lumbar segments [1]. Primary dehydration of intervertebral discs and the resulting reduction of the intervertebral space usually develop motor segment lowering, ligament slackening, annulus fibrosus protrusion, and ligament wrinkling and hypertrophy, which lead to lumbar degenerative diseases, such as lumbar disc herniation, lumbar spinal canal stenosis, lumbar spondylolisthesis [2, 3]. Lumbar degenerative spondylolisthesis (LDS) with the clinical manifestation of low back pain, sciatica, and neurogenic claudication, which often need decompression and fusion surgery to deal with the herniated disc and unstable alignment, in order to enlarge canal area, release the nerve root and improve the biomechanical condition. Since posterior lumbar interbody and fusion (PLIF) was reported by Cloward in 1943 [4], this procedure has performed widely all over the word along with the development of electrocoagulation, pedicle screw and interbody fusion cage. From 2002 to 2009, compared with cervical spine and thoracic spine, the annual operation rate of lumbar interbody fusion was the highest, increasing from 45 cases per 100,000 cases in 2002 to 72 cases per 100,000 cases in 2009 [5]. Joseph A analyzed 95,647 patients with LDS within Medicare beneficiaries from 2000 to 2011, 19,697 (21%) patients of which received decompression or fusion surgery [6]. Up to now, PLIF has been a general surgical procedure used by orthopedic surgeons to treat LDS.

The loss of naturally mobile vertebral segments after fusion may lead to an increased transmission of forces to adjacent non-fused segments, and significant compensatory increases in motion or micromotion at adjacent levels subsequent to increased stiffness and higher loads during normal activity [7, 8]. Cadaver studies have shown that with the increase of motion of adjacent segments after fusion, the intervertebral stress of adjacent segments increases [9, 10]. The incidence of radiological adjacent level disease (ALD) may be as high as 100% and clinical ALD as high as 27.5%, which indicates that there are many pathological changes but few symptoms [11]. The risk factors of ALD after PLIF procedure may include age, smoking status, primary degeneration, fusion mode and fusion segment length [12–14]. The application of spino-pelvic sagittal plane parameters and its relationship with postoperative clinical follow-up outcomes is increasingly emphasized in the recent reported studies, and sagittal balance plays a more and more important role in spinal surgery and preoperative planning [15, 16]. Key parameters of spine including pelvic incidence (PI), pelvic tilt (PT), sacral slope (SS), and spinal curvature, especially lumbar lordosis (LL) and segment lordosis (SL), were used to assess and analyze global sagittal plane balance.

Pedicle screws show high biomechanical strength, which are used to fix the spine through three columns. Through clinical treatments in recent years, we have found that the depth of pedicle screw insertion into the vertebral body in PLIF generally accounts for more than half of the anterior and posterior diameter of the vertebral body, and the longest depth even reaches the anterior wall of the vertebral body. We hypothesized that in PLIF, the depth of pedicle screw insertion can affect the long-term stability and sagittal balance of the lumbar spine. However, few studies have reported the effect of pedicle screw insertion depth on the sagittal parameters for the treatment of LDS. Therefore, this retrospective comparative study aimed to compare the effects of different pedicle screw insertion depths on sagittal balance and clinical prognosis of patients with LDS after receiving single-level PLIF.

## Methods

### Selection criteria

Inclusion criteria: (1) patients with LDS; (2) patients with intolerable low back pain or other neural symptoms resulting from compression; (3) patients with single-level PLIF; (4) patients were followed up for at least one year in the same designated hospital.

Exclusion criteria: (1) patients with previous fractures or surgical interventions at the spinal alignment; (2) LDS with tumor or tuberculosis; (3) patients who died or were unable to complete 12 months of follow-up.

### General information

According to the inclusion and exclusion criteria, a total of 88 patients treated with single-level PLIF for LDS from January 2018 to December 2019 were enrolled in this retrospective study. Finally, 52 patients were divided into long screw group (Group L) because the anterior edge of screws were more than 80% of the anteroposterior diameter of vertebral body, and 36 patients were divided into short screw group (Group S) because the anterior edge of screws were less than 60% of the anteroposterior diameter of vertebral body. The demographic data of patients in the two groups, included age, gender composition, surgical segment, bone mineral density (BMD), length of stay (LOS) and follow-up time.

### Surgical procedure

All operations in this series were performed by the same surgeon. All patients were placed in the prone position, intubated under general anesthesia, and the abdomen was suspended with the pelvis and manubrium sternum supported by pads. The entry point of the affected segment was confirmed with the assistance of fluoroscopy, and the osteophyte or dermobone at the entry point was removed. Under fluoroscopy observation, the opening device was inserted into the vertebral body along the pedicle, and the positioning needle was placed. The corresponding lamina and interlaminar ligamentum flavum were removed to expose the disc. The annulus fibrosus and the intervertebral disc were cut open, and the upper and lower cartilaginous endplates of the intervertebral disc were scraped with a ring curette. After removal of the disc tissues and endplate preparation, rods were placed bilaterally and pedicle screws in the slipped vertebrae were lifted to reduce forward slippage. Pedicle screws for sliding vertebrae used long tail screws. Through the locking of the nut, the sliding vertebrae was lifted and pulled to the unified level of the upper and lower vertebral bodies as far as possible. Distraction and posterior translation forces were applied while gradual slip reduction was achieved. The extent of slip reduction was verified with fluoroscopy. The intervertebral fusion device of an appropriate type was used, and the fusion device was filled with autologous bone fragments to implant the vertebral space, and screws and titanium rods (Medtronic Sofamor Danek, Memphis, TN, USA) were inserted for fixation in compression mode. Drainage tubes were placed and the incisions were closed one by one. Antibiotics were given for 3 days postoperatively to prevent infection.

### Radiographic evaluation

**Fig. 1:**
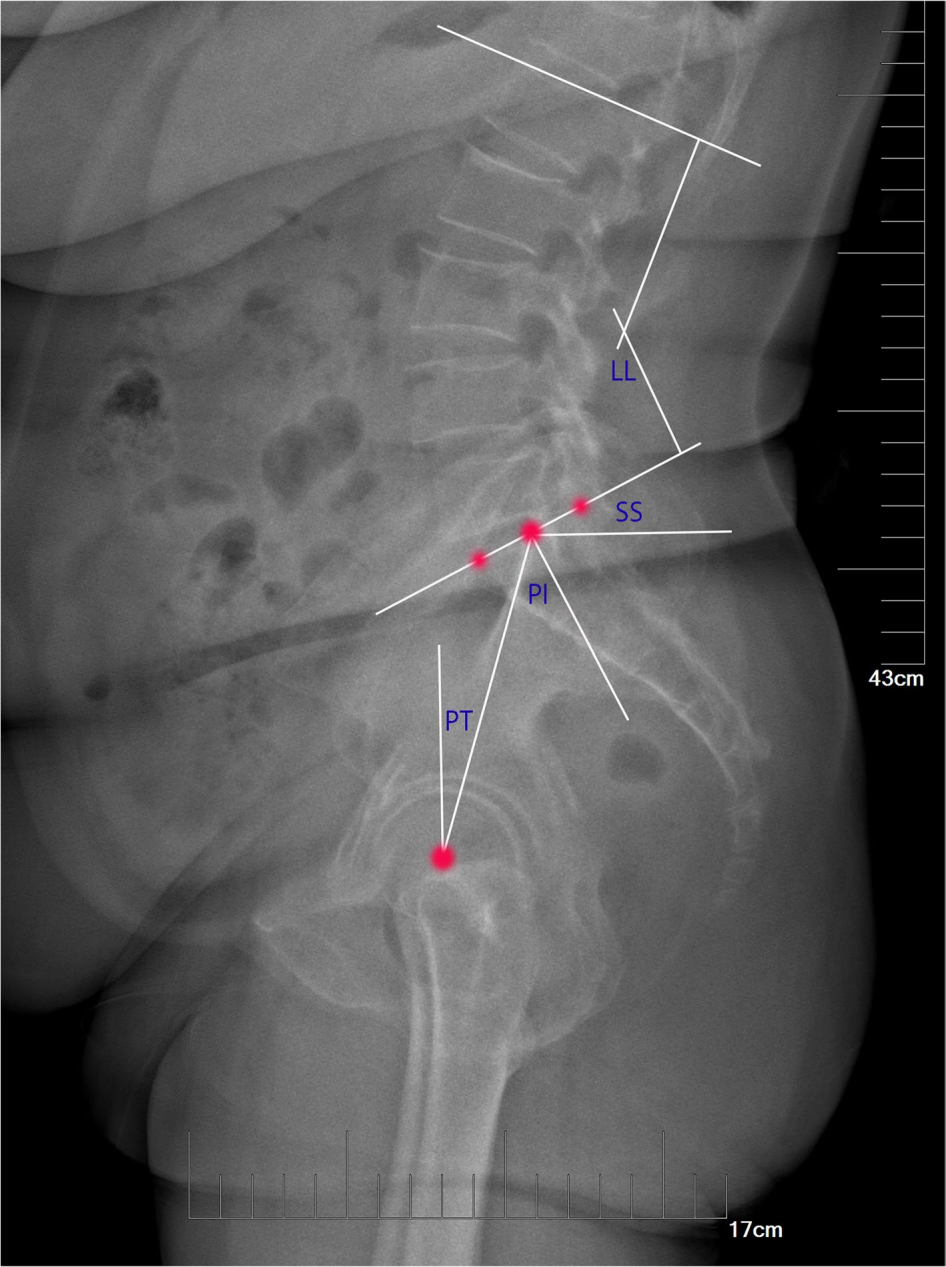
Plain lateral radiographs for measuring spino-pelvic sagittal plane parameters. LL: Lumbar lordosis; SS: Sacral slope; PI: Pelvic incidence; PT: Pelvic tilt

**Fig. 2:**
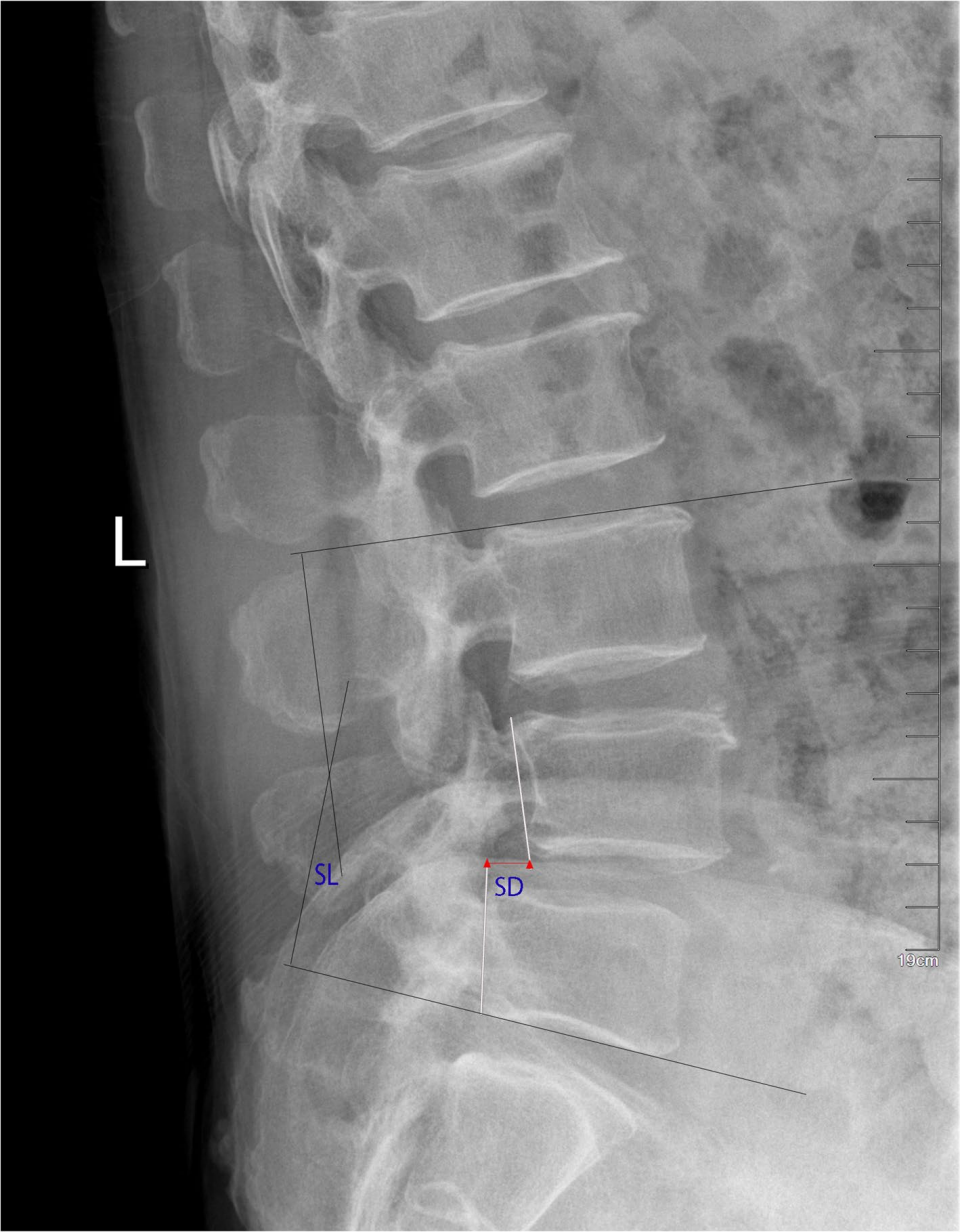
Plain lateral radiographs for measuring local deformity sparameters of spondylolisthesis. SD: Slip degree; SL: Segment lordosis

All radiological parameters were measured by two spinal surgeons (Figs. 1 and 2). The evaluation was conducted by blind method. Two observers measured each radiographic parameter of the same patient twice, and the data difference of each parameter was less than 5%, indicating that the measurement of the two observers has stability and reliability. The mean value of the four results measured for each parameter was used for analysis. The following radiographic parameters were measured [17]: Slip degree (SD), the distance between the two extended lines of the posterior aspect of upper and lower vertebrae. LL, the angle between the lines parallel to the superior endplate of S1 and the superior endplate of L1 vertebrae; SL, the angle between the upper endplate of the upper vertebrae and the lower endplate of the lower vertebrae of the responsible vertebrae; SS, the angle between the line parallel to the sacral plate and the horizontal line. PI, the angle between the line perpendicular to the midpoint of the sacral plate and the line connecting the midpoint of the femoral heads to the midpoint of the sacral plate. PT, the angle between the vertical line of the line between the midpoint of the sacral plate and the axis of the femoral heads.

### Functional evaluation

The patients filled out the following questionnaires before surgery, one month after surgery, and at the final follow-up: Oswestry Disability Index (ODI), and Visual Analog Scale (VAS) for back pain. The improvement of patients’ quality of life was assessed by ODI score, and subjective pain perception of patients was evaluated by VAS score (0–10 score, 0 indicated no pain, 10 indicated the most severe pain) [18].

### Statistical methods

SPSS 26.0 statistical software (SPSS Inc. Chicago, IL) was used for data analysis. The measurement data is expressed as mean ± standard deviation. Paired sample T test was used for comparison in the same group. χ^2^ test was used for categorical variable data. Multiple linear regression was used to find correlations between various results. *P* < 0.05 was considered statistically significant.

## Results

### Demographics

The demographic data of both groups were shown in Table 1. Among the patients included in this study, the average age was 59.83 ± 9.08 years old. Female patients (56) were more than male patients (32), and L5-S1 (47) was more common than L3–4 (7) and L4-L5 (34) in terms of the surgical segment. The mean BMD value of Group L (− 1.92 ± 0.61) was slightly lower than that of Group S (− 1.85 ± 0.55), but the difference was not statistically significant (*P* > 0.05). The average LOS was 13.11 ± 2.37 days, and the average follow-up time was 24.33 ± 6.27 months. There were no significant differences between the two groups in terms of age, gender, BMD, surgical segment, LOS and follow-up time (*P* > 0.05).

**Table 1:** Demographic data of patients in the two groups

	Full sample	Group L	Group S	*P* value
Number of patients	88	52	36	
Age (years)	59.83 ± 9.08	58.81 ± 9.57	61.31 ± 8.22	0.206
Gender (male/female)	32/56	18/34	14/22	0.682
BMD (T-score)	−1.89 ± 0.58	− 1.92 ± 0.61	−1.85 ± 0.55	0.568
Surgical segment (n)				
L3-L4	7	4	3	0.921
L4-L5	34	21	13	
L5-S1	47	27	20	
LOS (days)	13.11 ± 2.37	12.85 ± 2.52	13.50 ± 2.12	0.205
Follow-up (months)	24.33 ± 6.27	23.29 ± 6.74	25.83 ± 5.26	0.061

BMD: bone mineral density; LOS: length of stay

### Radiographic outcomes

**Fig. 3:**
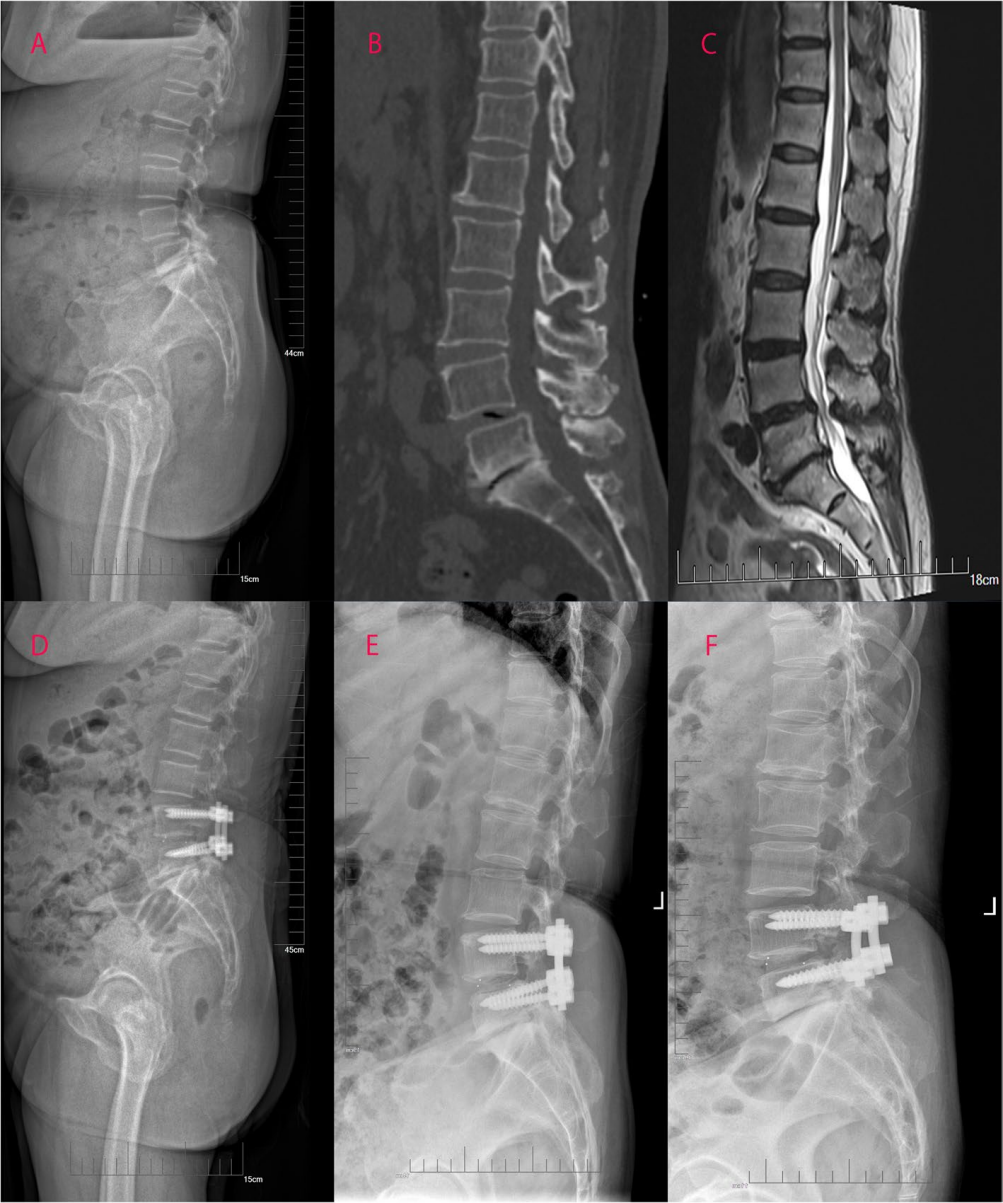
Preoperative sagittal lateral view (A), sagittal-computed tomographic scan (B), sagittal T2-weighted magnetic resonance image (C), sagittal lateral view one month after surgery (D and E) and sagittal lateral view at the final follow-up (F) of a 67-year-old female patient with L4 degenerative spondylolisthesis treated with PLIF with long screw fixation

**Fig. 4:**
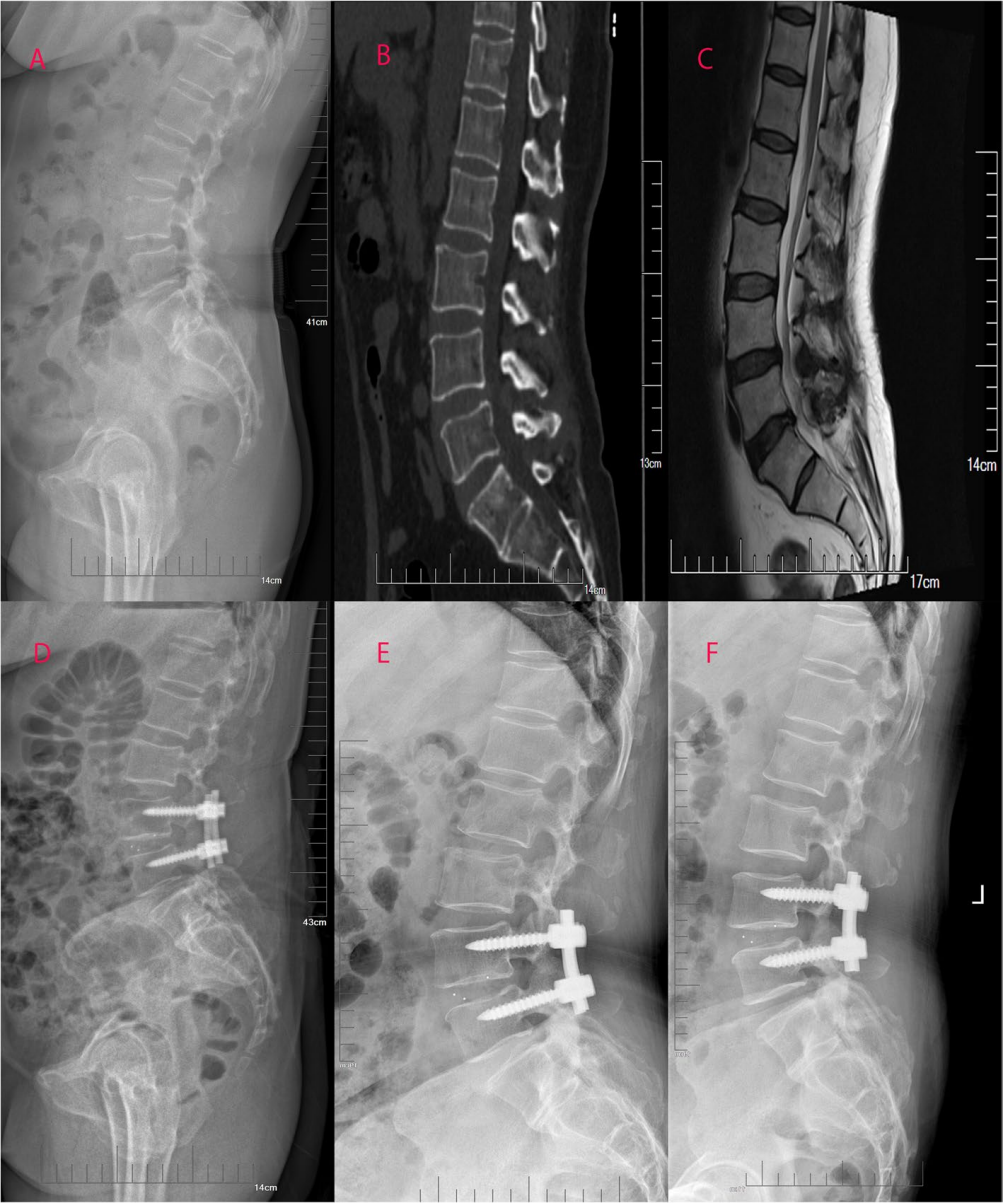
Preoperative sagittal lateral view (A), sagittal-computed tomographic scan (B), sagittal T2-weighted magnetic resonance image (C), sagittal lateral view one month after surgery (D and E) and sagittal lateral view at the final follow-up (F) of a 54-year-old female patient with L3 degenerative spondylolisthesis treated with PLIF with short screw fixation

The radiographic parameters of patients in the two groups were shown in Table 2. Except that PI in Group S at the final follow-up was not statistically different from the preoperative value (*P* > 0.05), there were significant differences in other parameters one month after surgery and at the final follow-up compared with preoperative values (*P <* 0.05). For the comparison between Group L and Group S, none of the parameters differed significantly before and one month after surgery (*P* > 0.05). At the final follow-up, SD, SL, LL, PT and PI-LL differed significantly between the two groups (*P <* 0.05) (Figs. 3 and 4).

**Table 2:** Radiographic parameters of patients in the two groups

	Group L (*n* = 52)	Group S (*n* = 36)	*P* Value
SD (mm)			
PRE	17.56 ± 5.60	16.97 ± 5.14	0.620
1 MONTH	6.85 ± 2.97*	7.11 ± 3.22*	0.692
FINAL	8.00 ± 2.97*	9.47 ± 3.23*	0.002**
SL (°)			
PRE	16.36 ± 4.23	16.78 ± 4.72	0.669
1 MONTH	25.25 ± 2.84*	24.69 ± 3.20*	0.394
FINAL	25.77 ± 2.74*	23.69 ± 2.81*	0.001**
LL (°)			
PRE	39.08 ± 3.64	38.08 ± 3.61	0.210
1 MONTH	45.85 ± 4.09*	44.58 ± 4.01*	0.155
FINAL	45.50 ± 4.19*	43.36 ± 3.96*	0.018**
SS (°)			
PRE	36.35 ± 4.99	34.78 ± 4.72	0.142
1 MONTH	37.98 ± 4.27*	36.58 ± 4.22*	0.133
FINAL	38.15 ± 4.51*	36.39 ± 5.02*	0.089
PT (°)			
PRE	18.00 ± 4.91	18.83 ± 4.18	0.409
1 MONTH	14.87 ± 4.27*	15.69 ± 3.92*	0.357
FINAL	14.62 ± 4.97*	16.86 ± 5.35*	0.047**
PI (°)			
PRE	54.35 ± 3.55	53.61 ± 3.49	0.339
1 MONTH	52.85 ± 3.72*	52.28 ± 3.92*	0.493
FINAL	52.77 ± 4.20*	53.25 ± 4.78	0.619
PI-LL (°)			
PRE	15.27 ± 4.10	15.53 ± 2.78	0.743
1 MONTH	7.00 ± 4.51*	7.69 ± 3.82*	0.452
FINAL	7.27 ± 4.72*	9.89 ± 4.52*	0.011 **

SD: slip degree; SL: segment lordosis; LL: lumbar lordosis; SS: sacral slope; PI: pelvic incidence; PT: pelvic tilt. PRE: preoperative, 1 MONTH: one month after surgery, FINAL: final follow-up

* Significance compared with the preoperative, *P* < 0.05

** Significance between the two groups, *P* < 0.05

### Functional outcomes

The functional outcomes of patients in the two groups were shown in Table 3. Compared with the preoperative results, ODI and VAS one month after surgery and at the final follow-up all decreased significantly in both groups (*P <* 0.05). In addition, significant differences of ODI and VAS were found between Group L and Group S at the final follow-up (*P <* 0.05), although there was no significant difference before and one month after surgery (*P* > 0.05).

**Table 3:** Functional outcomes of patients in the two groups

	Group L (n = 52)	Group S (n = 36)	*P* Value
ODI			
PRE	67.88 ± 8.11	66.83 ± 7.40	.537
1 MONTH	26.75 ± 4.61*	26.06 ± 3.75*	.456
FINAL	25.87 ± 4.54*	28.08 ± 3.71*	.017**
VAS			
PRE	7.35 ± 0.95	7.22 ± 0.80	.522
1 MONTH	2.67 ± 0.90*	2.69 ± 0.89*	.913
FINAL	2.54 ± 0.67*	3.02 ± 0.77*	.002**

ODI: Oswestry Disability Index; VAS: Visual Analogue Scale. PRE: preoperative, 1 MONTH: one month after surgery, FINAL: final follow-up

* Significance compared with the preoperative, *P* < 0.05

** Significance between the two groups, *P* < 0.05

Multiple linear regression was performed to analyze the relationship between radiological parameters and ODI and VAS scores at the final follow-up (Supplementary Table). SD, SL, LL, SS, PI, PT and PI-LL at the final follow-up were included in this analysis. The results showed that SD, SL, LL and PI-LL showed positive correlation with ODI and VAS scores at the final follow-up (*P <* 0.05).

### Related complications

The related complications of the two groups were shown in Table 4. Patients in Group L and Group S had no complication during the surgical procedure. A total of 3 patients experienced complications during their hospitalization, but there was no statistically significant difference in the incidence of complications between the two groups (*P* > 0.05). In Group L, one patient with pneumonia on the fourth day after operation was treated with intravenous injection of the third-generation cephalosporin for one week, and one patient with deep venous thrombosis on the third day after operation was treated with pneumatic compression socks and low molecular weight heparin for one week, and this patient did not develop pulmonary embolism. In Group S, there was one patient who suffered from the superficial infection of the wound on the third day after surgery. For this patient, third-generation cephalosporin was injected intravenously for 7 days. All of the above patients were discharged from the hospital until their symptoms were completely gone. At the final follow-up, both groups showed good intervertebral fusion. The intervertebral fusion rate was 96.15% (50/52) in Group L and 94.44% (34/36) in Group S, with no statistically significant difference between the two groups (*P* > 0.05). Although there were 2 cases of pedicle screw loosening in Group S at the last follow-up, there was no statistical difference between the two groups (*P* > 0.05).

**Table 4:** Related complications of patients in the two groups

	Group L (n = 52)	Group S (n = 36)	*P* value
Hospitalization	Pulmonary infection (1); Deep vein thrombosis (1)	Superficial infection of the wound (1)	0.786
Final follow-up			
Intervertebral fusion rate	96.15% (2)	94.44% (2)	0.705
Screw loosening rate	0% (0)	5.56% (2)	0.086

## Discussion

Spondylolisthesis is considered an acquired disease that is relatively common but usually asymptomatic, and the prevalence increases with age [19]. Because of persistent and intolerable low back pain and other symptoms caused by nerve compression, some patients with LDS need surgery to relieve their symptoms. Over the years, the positive improvement effect of PLIF on spondylolisthesis and other lumbar degenerative diseases has been confirmed, and this surgical method has been widely used [20]. The biomechanical environment of lumbar interbody fusion is characterized by the presence of a rigid lever arm represented by the pelvis and sacrum, adjacent to a series of more active but lordotic motion segments of the lower lumbar spine [7]. Lumbar sagittal balance has been shown to be an independent predictor of clinical outcomes in surgical patients with adult spinal deformity, degenerative disc disease, and LDS. Several recent studies have not only confirmed that the main biomechanical features of patients with LDS are anterior translation of sagittal plane balance and loss of LL with an increase in PT [21], but also demonstrated restoring sagittal balance after surgery can improve long-term clinical outcomes and reduce the risks of sagittal imbalance such as adjacent segmental disease and screw loosening [22–24]. Therefore, in order to evaluate the effects of different screw depths on postoperative spino-pelvic sagittal balance in patients with LDS, key parameters such as SD, PI, PT, SS, LL, SL and PI-LL were used in this study to evaluate and analyze the global spinal sagittal balance.

Pelvic parameters are PI, PT and SS. PT, which is characteristic of pelvic rotation, decreases with forward inclination and increases with subsequent inclination [25]. The standard value of PT is about 13° ± 6° [26]. In the two groups of this study, the average preoperative PT were both about the upper limit standard, and PT decreased to about normal value one month after surgery and at the final follow-up. Some studies [27–29] have confirmed that the improvement of PT plays an important role in sagittal reconstruction, and is indicative of good clinical outcome, which can explain why both groups of patients in this study had a significant recovery in postoperative ODI and VAS. In addition, Kim et al. [29] found that patients with PT improvement show significantly better VAS and ODI scores than those without improvement. At the final follow-up, there was a statistical difference in PT between the two groups, which may also explain why there were differences in ODI and VAS between the two groups. PI, as an individual variable independent of body position, increased from age 4 to 18 but did not change further into adulthood, which also allowed PI to define the position of the pelvis and all other pelvic parameters (PT, SS) to be adjusted accordingly [25, 30]. For example, LL depends on the size of PI. If PI value is higher, both SS and LL will increase, and vice versa. The standard value of PI is approximately 53° ± 9° [31]. SS is defined as the angle between the horizontal line and the line parallel to the sacral plate, which is approximately 41° ± 8°. PI, which is not affected by posture, can be used as an indicator to describe the shape of pelvis and sacrum orientation since the above three pelvic parameters fulfill the equation: PI = PT + SS [32].

The LL is measured by the Cobb method, which is the angle between the lines drawn parallel to the superior endplate of L1 and the superior endplate of S1. The standard value of LL is approximately 46.5° [32, 33]. There is a close relationship between LL and PI. In general, the extent of LL depends on the value of PI, and the ideal formula is: LL = PI ±9°. If these two parameters do not match, it will cause the imbalance of sagittal balance of lumbar spine. Therefore, in recent years, a new parameter, PI-LL, has been produced between PI and LL, which can more directly quantify the mismatch between pelvis shape and lumbar curve, so it can be used to guide the lumbar surgery plan and the recovery target of patients after surgery [34]. One of the goals of spine pelvis sagittal alignment is that PI-LL < 10° threshold [35]. In this study, both groups of patients with LDS showed that LL was lower than the minimum standard and PI-LL could not reach the ideal standard before surgery. After patients experienced PLIF, LL and SL in both groups increased significantly and PI-LL also decreased to the target range. These results are mainly attributed to the fact that PLIF can effectively restore vertebral height, increase segmental stability and maintain normal sagittal balance parameters of lumbar spine and pelvis through bone grafting and fusion [36].

An important advantage of reduction of the vertebral slippage is that it can correct sagittal deformity of spine, which is indirectly conducive to better nerve root decompression and better opportunity to obtain fusion. In a study reported by Wegmann et al. [37], they concluded that reducing vertebral SD during PLIF was positively correlated with clinical outcomes 12 and 24 months after surgery. In this study, SD in both groups after PLIF was significantly improved compared with that before surgery. It was found that there was no significant difference between the two groups in the reduction of SD after PLIF, but Group L could better maintain the reduction of SD at the final follow-up. Further, the above results were consistent with the clinical outcomes of the two groups, which confirmed the conclusion of Wegmann et al. to a certain extent.

Previous studies have shown that the insertion depth, number and angle of pedicle screws can significantly affect the biomechanical stability of the screws, thus affecting the loading and stiffness of the fixed segment of the spine [38–40]. However, in clinical lumbar surgery, the angle, number and depth of pedicle screw placement are partly affected by the treatment experience of spinal surgeons for different patients, and this empirical judgment sometimes confuses spine surgeons. All operations in this study were performed by the same surgeon. Although the same surgeon may have a relatively fixed surgical procedure, such as a preference for long screws, individual differences in each patient lead to differences in the length of screws placed during surgery. While, the skill of the surgeon was a relatively stable factor and therefore could not account for the difference in outcomes between the two groups. Insufficient depth of pedicle screw insertion may seriously affect the riveting strength between the screw and the vertebral body, while too long pedicle screw may penetrate the anterior cortex of the vertebral body, thus increasing the risks of injury to adjacent structures [41]. In a cadaver study conducted by Kristophe et al. [38], they concluded that increased insertion depth will result in enhanced screw-bone purchase leading to lower screw loosening and greater pullout strength, but this effect is only significant in bicortical screws. However, Matsukawa et al. [42] studied pedicle screw fixation strength in osteoporotic vertebrae and pointed that longer screws increased the degree of bone contact, which may have contributed slightly to the increased pullout strength even without engagement with the anterior cortex. And they also pointed out that deeper screw insertion and the use of a larger diameter screw were reasonable for the maintenance of stability until adequate bone arthrodesis was achieved. In addition, Oe et al. [43] biomechanically demonstrated that a higher occupancy rate of pedicle screw decreases the load on the vertebral body, and presumed that the longer the screws, the higher the stability. In this retrospective study, we routinely tested the BMD of patients before surgery and the average values of both groups showed poor bone quality. On the basis of no statistical difference in BMD values, we analyzed the influence of different screw depths on the prognosis of patients through spino-pelvic sagittal balance parameters. At the final follow-up, we noted significant differences in SL, LL, PT and PI-LL between the two groups of patients, suggesting that short screws may not be as effective as long screws in providing adequate fixation strength and maintaining good spino-pelvic sagittal balance for postoperative patients. Further, multiple linear regression analysis showed a positive relationship between ODI and VAS scores and some radiological parameters at the fianl follow-up. The reason for the above results may be that after a long period of activity of the spine, due to the short length of pedicle screws, the riveting force with the vertebral body may be not strong, and the load on the vertebral body may be increased, which may affect the stability of the spine to some extent and lead to changes in some parameters of sagittal balance. Therefore, we suggest that for patients with LDS requiring single-level PLIF, the depth of the pedicle screws into the vertebral body can be appropriately increased while ensuring that the depth of the pedicle screws is within the safe range.

This study had several limitations. First, it was designed as a retrospective comparative study, and the sample size was relatively insufficient, especially in the short screw group. Second, the follow-up time of some patients was relatively short, and we could not arbitrarily conclude that the main reason for the difference in clinical results between the two groups is the difference in radiological parameters between the two groups, because this needs a longer follow-up time to analyze and verify. Third, this study did not study deeply the risk factors that that affected the spino-pelvic sagittal plane parameters in both groups. Finally, BMD was measured for all enrolled patients, but we did not group them based on whether they had osteoporosis or not, which may affect the long-term effects in both groups. Therefore, future studies may require a prospective randomized controlled study and a longer follow-up time to further analyze whether the differences in radiological parameters between the two groups are closely consistent with the differences in clinical outcomes. In addition, patients with different BMD values need to be further grouped in order to prevent patients with osteoporosis and patients with normal bone mass mixed together.

In summary, PLIF is a clinically safe and effective method for LDS. Through effective decompression, fixation and fusion, patients with LDS are able to restore the spine to the desired biomechanical structure to some extent, thereby improving their quality of life. Through this study, in the clinical treatment of patients with single-level PLIF, we can provide a strong evidence that, within a certain safety range, spinal surgeons can choose to use long screws as much as possible, which is conducive to the recovery and maintenance of the spino-pelvic sagittal balance.

## Conclusions

Regardless of the depth of the screw, PLIF can significantly improve the clinical efficacy of patients with LDS. However, in terms of outcomes with an average follow-up time of 2 years, the deeper the screw depth is within the safe range, the better the spino-pelvic sagittal balance may be restored and the better the quality of life may be.

## Supplementary Information

**ESM 1.**

## Data Availability

Not applicable.

## Abbreviations

PLIF: posterior lumbar interbody and fusion
LDS: lumbar degenerative spondylolisthesis
PI: pelvic incidence
PT: pelvic tilt
SS: sacral slope
LL: lumbar lordosis
SL: segment lordosis
SD: slip degree
ODI: Oswestry Disability Index
VAS: Visual Analog Scale
ALD: adjacent level disease
BMD: bone mineral density
LOS: length of stay
