# Supplementary material for: Effects of different pedicle screw insertion depths on sagittal balance of lumbar degenerative spondylolisthesis, a retrospective comparative study
Source: BMC Musculoskelet Disord. 2021 Oct 5;22:850. doi: 10.1186/s12891-021-04736-1 (PMC8493756; doi:10.1186/s12891-021-04736-1)
Supplement: Supplementary file 1 — ESM 1. [file 12891_2021_4736_MOESM1_ESM.docx]

| **Supplementary table. Multivariate linear regression analysis of parameters associated with ODI and VAS score at the final follow-up.** | | | | |
| --- | --- | --- | --- | --- |
| Parameters  (At the final follow-up) | ODI | | VAS | |
|  | Standardized coefficient | P | Standardized coefficient | P |
| SD | 0.285 | 0.004* | 0.277 | 0.005* |
| SL | -0.233 | 0.016* | -0.266 | 0.005* |
| LL | -0.295 | 0.010* | -0.309 | 0.006* |
| SS | -0.084 | 0.415 | -0.076 | 0.423 |
| PI | 0.190 | 0.140 | 0.192 | 0.127 |
| PT | 0.092 | 0.415 | 0.076 | 0.493 |
| PI-LL | 0.289 | 0.009* | 0.482 | 0.000* |
| SD: slip degree; SL: segment lordosis; LL: lumbar lordosis; SS: sacral slope; PI: pelvic incidence; PT: pelvic tilt.  * represents statistically significant, *P*<0.05. | | | | |
